# Supplementary material for: Identifying repeat domains in large genomes
Source: Genome Biol. 2006 Jan 31;7(1):R7. doi: 10.1186/gb-2006-7-1-r7 (PMC1431705; doi:10.1186/gb-2006-7-1-r7)
Supplement: Additional File 1 — A zipped file of browsable HTML files with a complete list of the connected components in the repeat domain graph of human Repbase. [file gb-2006-7-1-r7-S1.gz › html/subgraphs/8688.pdf]

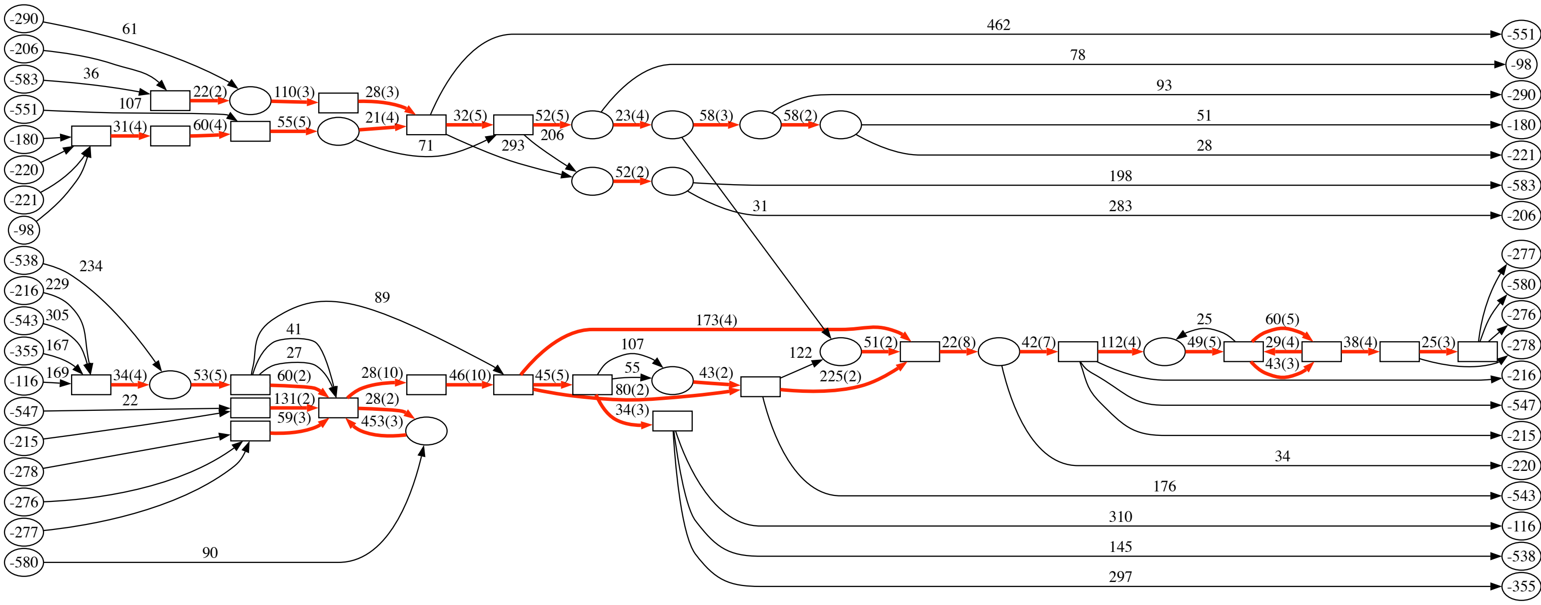

-290
-206
-583
-551
-180
-220
-221
-98
-538
-216
-543
-355
-116
-547
-215
-278
-276
-277
-580
61
36
107
22(2)
110(3)
28(3)
462
78
93
51
28
198
283
31(4)
60(4)
55(5)
21(4)
71
32(5)
293
52(5)
206
23(4)
58(3)
58(2)
52(2)
31
234
229
305
167
169
34(4)
53(5)
89
41
27
60(2)
131(2)
59(3)
22
28(10)
28(2)
453(3)
46(10)
45(5)
173(4)
107
55
80(2)
43(2)
34(3)
122
51(2)
225(2)
22(8)
42(7)
112(4)
25
49(5)
60(5)
29(4)
43(3)
38(4)
25(3)
34
176
310
145
297
90
-551
-98
-290
-180
-221
-583
-206
-277
-580
-276
-278
-216
-547
-215
-220
-543
-116
-538
-355
